# Investigation of Bonding Behavior of FRP and Steel Bars in Self-Compacting Concrete Structures Using Acoustic Emission Method

**DOI:** 10.3390/s19010159

**Published:** 2019-01-04

**Authors:** Bo Di, Jingkai Wang, Haotian Li, Jinhang Zheng, Yu Zheng, Gangbing Song

**Affiliations:** 1 School of Environment and Civil Engineering, Dongguan University of Technology, Dongguan 523808, China; dibo@dgut.edu.cn (B.D.); 2172332355@email.szu.edu.cn (H.L.); 201641201307@dgut.edu.cn (J.Z.); 2 State Key Laboratory of Coastal and Offshore Engineering, Dalian University of Technology, Dalian 116024, China; wjk@mail.dlut.edu.cn; 3 College of Civil Engineering Shenzhen University, Shenzhen University, Shenzhen 518060, China; 4 Department of Mechanical Engineering, University of Houston, Houston, TX 77204-4006, USA; gsong@uh.edu

**Keywords:** acoustic emission, FRP, self-compacting concrete, bonding, pull-out test

## Abstract

To extend understanding of the bonding behavior of fiber reinforced polymer (FRP) and steel bars in self-compacting concrete (SCC), an experimental series consisting of 36 direct pull-out tests monitored by acoustic emission (AE) were performed in this paper. The test variables involved rebar type, bar diameter, embedded length, and polypropylene (PP) fiber volume content. For each test, the pull-out force and free end slip were continuously measured and compared with the corresponding AE signals. It was found that the proposed AE method was effective in detecting the debonding process between the FRP/steel bars and the hosting concrete. The AE signal strength exhibited a good correlation with the actual bond stress-slip relationship measured in each specimen. Based on the AE location technique, the invisible non-uniform distribution of bonding stress along the bar was further revealed, the initial location of damage and the debonding process were captured. Additionally, the contribution of bar-to-concrete load-bearing mechanism (chemical adhesion, friction, and mechanical interlocking) to sustain the pull-out force was effectively clarified by studying the collected signals in the frequency domain of AE methods. The experimental results demonstrate that the proposed AE method has potential to detect the debonding damage of FRP/steel bar reinforced SCC structures accurately.

## 1. Introduction

High-performance and environment-friendly materials are currently attracting a widespread attention in civil engineering. High strength, light weight, long-term durability, and low maintenance costs are some remarkable advantages of these new materials compared to traditional ones. Self-compacting concrete (SCC) is a new type of concrete material, which owes excellent workability and high resistance to segregation. Due to its high degree of flowability, no vibration equipment is needed for the compacting procedure. This material is a technically viable substitute for normal concrete (NC) and has gained wide use in different applications [1,2]. Currently, fiber reinforced polymer (FRP) has been recognized as a construction material [3,4,5,6] and can be used as an alternative reinforcement for concrete structures due to its high strength, light weight, and high corrosion resistance [7,8]. With the advantages of FRP and SCC, combining them would provide a promising solution in the construction projects [9]. However, the application of FRP bars as internal reinforcement in SCC structures is yet in its early stages, and the mechanical properties should be further studied. A relevant behavior problem is the bond between FRP composites and SCC, since it is a critical factor that influences the structural performance under both service and ultimate conditions [10]. In addition, with the recent emphasis on structural health monitoring [11,12,13] and damage detection [14,15,16], the monitoring of bonding behavior is of great importance to enhance the safety level and to widen the application of FRP reinforced SCC structures [17,18,19,20].

Many studies have been conducted to investigate the bonding behavior between FRP/steel bars and normal concrete [21,22,23,24,25,26,27,28,29,30,31]. The key factors affecting bonding performance, such as rebar type, bar diameter, embedment length, confinement pressure, and concrete strength, have been studied based on either direct pull-out test or beam test [21,22,23,24,25,26,27,28,29,30,31,32,33]. Baena et al. [21] carried out an experimental program consisting of 88 pull-out specimens by using carbon fiber reinforced polymer (CFRP) and glass fiber reinforced polymer (GFRP) bars, and these bars presented a bonding strength lower than the steel bars. Based on the results from previous studies, the bonding strengths of specimens with FRP bars were typically lower than conventional steel bars. On the contrary, the slip of FRP bars relative to the surrounding concrete was usually greater than that of steel bars [21,22,23]. Moreover, a trend of decreasing bonding strength with increasing bar diameters can be observed in the literature [21,24,25,26]. The major reasons for this trend could be the Poisson effect [21], the shear lag effect [24,25], and the size effect [26]. By installing a strain probe inside the pre-drilled FRP bar, Alzahrani et al. [27] observed the nonlinear distribution of bond stress along the embedded length. It was found that doubling the embedment length reduced the average bonding strength by 25%. The dependence of bond strength on embedment length could be explained by the nonlinear stress distribution, since the nonlinear stress distribution is more evident in the case of the larger embedment length, which results in the lower average bonding strength [28,29]. Furthermore, some experimental and theoretical investigations have been carried out on the bond behavior of FRP/steel bars in SCC structures [32,33,34,35]. Mazaheripour et al. [32] conducted beam tests to study the bonding performance between GFRP bars and steel fiber reinforced self-compacting concrete. It was concluded that concrete cover and bonding length played an important role on the bonding strength of GFRP bars. The high fiber contents contributed to a better bonding behavior by reducing crack width in the concrete cover, which resulted in an increase in the average residual bond stress. It should be mentioned that the most commonly used bond stress-slip curves acquired by direct pull-out tests or bending tests are the macro-reflection of the bond between reinforcement bars and concrete. The advanced measuring methods should be adopted to further reveal the bar-to-concrete load-bearing mechanism and the debonding damage process.

Piezoceramic transducers have the advantages of both sensing and actuation capacities [36,37], high bandwidth [38,39], and low cost, and have been actively studied to monitor debonding problems in various structures [40,41,42,43], including rebar and concrete debonding [44,45,46]. In addition, in the form of a smart aggregate, a piezoceramic transducer can be easily and reliably embedded in a concrete structure [47,48], or in the form of a patch, a piezoceramic transducer can be surface-bonded on a structure of interest [49,50], including a steel rebar or a composite rebar. Jiang et al. proposed an innovative method using piezoceramic transducers and wavelet packet analysis to detect the debonding between an FRP bar and the concrete structure [18]. Xu et al. developed a novel piezoceramic-based active sensing approach to monitor the debonding between a GFRP bar and the concrete structure, and experimental results demonstrated the effectively of the proposed approach [44]. With the help of piezoceramic transducers and the hierarchical clustering analysis, Sevillano et al. identified interfacial crack-induced debonding in FRP reinforced concrete beams [46].

Utilizing the high bandwidth of piezoceramic transducers, the acoustic emission (AE) technique, a passive monitoring method, is often used for damage detection, including debonding monitoring. Compared to other sensing methods, the AE technique collects the transient elastic waves caused by the rapid release of energy in the process of materials fracture. By analyzing these transient elastic waves, the damage degree of materials can be investigated and evaluated. This technique has been increasingly used in civil engineering [51,52], mechanical engineering [53,54], and others [55,56]. Bunnori et al. [57] applied the AE technique to detect the early cracks in concrete beams and found that the AE parameters were sensitive to the initiation and the growth of cracks. Aldahdooh et al. [58] classified the types of cracks (flexural or shear cracks) of RC beams subjected to four-point bending by AE technique. Moreover, some researches applied the AE technique to monitor the debonding process of steel bars in concrete structures. Balazs et al. [59] applied the AE technique to monitor the damage accumulation on deformed steel bar to concrete interaction and confirmed that the AE parameters were consistent with the test phenomena. Gallego et al. [60] utilized the AE signals obtained from the pull-out tests to compare the performance of black steel and hot-dip galvanized steel. It was revealed that it was possible to identify the transitional points of pullout force–slip curves by measuring the AE activity. Abouhussien et al. [61,62] found that the cumulative number of hits and cumulative signal strength were in a good relationship with the different damage degree of debonding process, and proposed the developed intensity classification charts based on AE intensity analysis. Furthermore, Wang et al. [63] conducted a series of pull-out tests to study the bond behavior between corroded steel bars and concrete based on the AE method. The test results showed that the AE location technique could be used to detect the actual crack development, and the characteristics of the AE signal reflected the bonding behavior of the pull-out specimens.

Nevertheless, the AE signals obtained from pull-out tests have not been utilized to evaluate the bonding behavior of FRP bars in SCC structures. This paper presents an experimental investigation aimed at clarifying the load-bearing mechanism and debonding damage process between FRP/steel bars and SCC. The pull-out specimens with variable rebar types, bar diameters, embedded lengths, and concrete mixtures were designed and fabricated. The bonding behavior is identified in terms of the stress-slip curves and the AE signals measured by two AE sensors attached at the end faces of the bar. Test results indicate that the proposed AE method can be used to detect the debonding process of FRP/steel bars in SCC structures.

## 2. Experimental Program

### 2.1. Material Properties

#### 2.1.1. Reinforcement Bars

**Figure 1:**
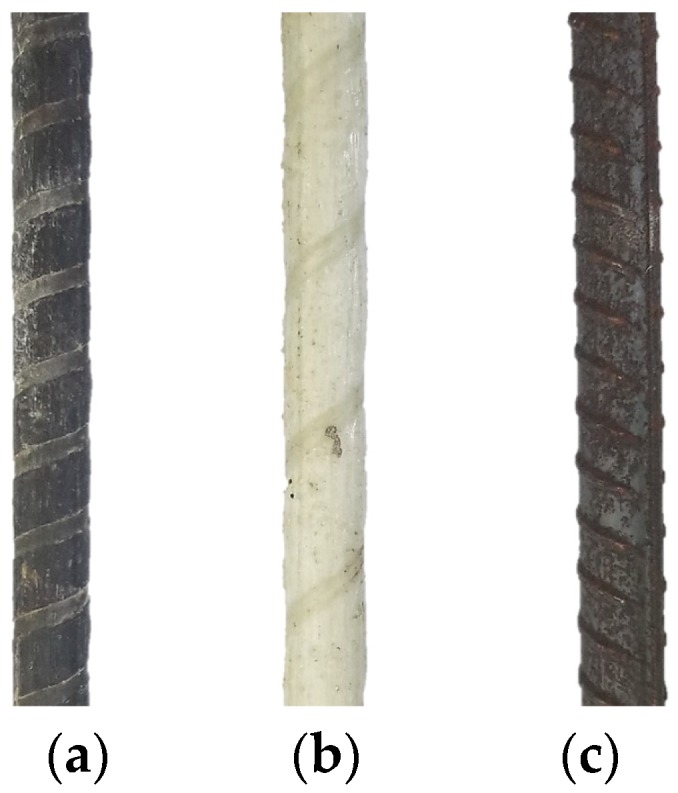
Reinforcement bars: (**a**) BFRP bar; (**b**) GFRP bar; (**c**) Steel bar.

Three types of reinforcing materials were used in the study, which included basalt fiber reinforced polymer (BFRP), GFRP, and steel bars. The surface treatment and characteristics of the bars used are shown in Figure 1. BFRP bars with a textured surface and GFRP bars with a helically wrapped and sand-coated surface were adopted, respectively. On the other hand, ribbed steel bars were used for comparison purposes. As shown in Table 1, two diameters, 12 and 20 mm, were considered for BFRP bars, while only one diameter (12 mm) was adopted for GFRP and steel bars. The geometrical and mechanical properties of the reinforcement bars are listed in Table 1.

#### 2.1.2. Self-Compacting Concrete

In this investigation, SCC was used in the direct pull-out specimens. To examine the influence of fiber content on the bonding behavior of FRP/steel bars, three different fiber volume contents, 0.0%, 0.3%, and 0.6%, were adopted. The concrete used for the pull-out specimens was prepared in the laboratory and its compositions are shown in Table 2. Ordinary Portland cement labeled as 32.5R, fine river sand and crushed gravel aggregate with maximum size of 20 mm were used. Fly ash with fineness of 250 meshes was adopted to replace cement for up to 50% by weight to produce the SCC. Polypropylene fibers (PP fibers) of 12 mm length and 18 um diameter were used. The SCC mixture showed good workability and cohesion, and the total spread measured in the slump-flow tests ranged from 720 and 800 mm, without deposition and segregation.

For each batch of concrete mix, three standard cube samples (150 × 150 × 150 mm) and two cylinder samples (300 × 150 mm) were cast and cured for 28 days under the same conditions as the specimens. The concrete compressive strength and tensile strength were determined based on the standard cube samples. Additionally, the cylinder samples were used to determine the Young’s modulus of the concrete. The mechanical properties of the SCC used for the pull-out specimens are listed in Table 3.

### 2.2. Details of Test Specimens

**Figure 2:**
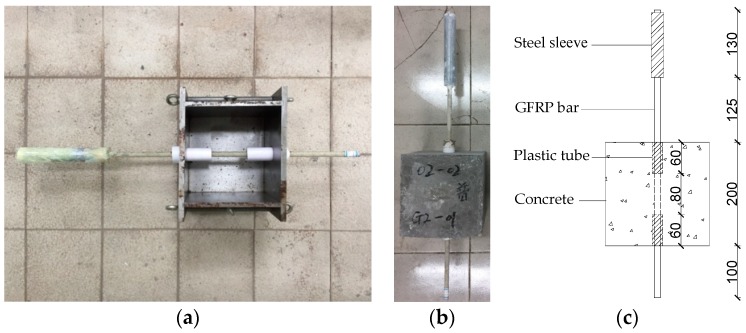
Pull-out specimen: (**a**) Metal mould; (**b**) Final specimen; (**c**) Specimen dimensions (mm).

A series of experimental test consisting of 36 direct pull-out tests using AE were performed to investigate the bonding behavior of FRP/steel bars in SCC. Concrete cubes sized 200 × 200 × 200 mm with different types of bars, bar diameters, embedded lengths, and fiber contents, were designed and fabricated. Some metal moulds were used for casting the concrete cubes. The bars were installed vertically in the moulds before casting, lying in the central portion of the specimens, as shown in Figure 2a. To avoid fiber damage at the gripping zone, a special protection system consisting of steel sleeve and expansive cement was adopted at the loaded end of rebars. Moreover, plastic tubes without bonding requisites to cement-based materials were adopted to cover the bars for providing non-contact areas between concrete and bars. The bonded areas were positioned in the middle of the concrete cubes for all specimens, and the embedded length *L*_e_ was changed from 40 mm to 120 mm, as shown in Figure 2c.

After 24 h of casting, all the specimens were demoulded and then cured in a natural indoor environment at a temperature of 25 ± 3 °C and a humidity of about 95% for 28d. Figure 2b shows the final specimen reinforced with GFRP bar of 12 mm diameter (Φ12) and with an embedment length of 80 mm

### 2.3. Test Setup and Loading Procedure

**Figure 3:**
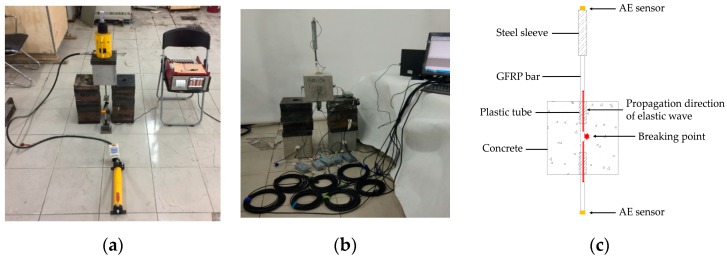
The direct pull-out test system: (**a**) Loading device and displacement measurement equipment; (**b**) AE acquisition system setup; (**c**) Locations of the AE sensors.

The test setup configuration is shown in Figure 3a, where a hydraulic driving anchor puller, with a maximum load capacity of 100 kN and a maximum stroke of 60 mm, was used to provide the pull-out force. The specimens were loaded under an incrementally increasing load condition until bonding failure, the increasement of the load was taken as 0.5 kN. The free end slip of the bar was measured using one linear variable differential transformer (LVDT) mounted at the bottom of each specimen. One load cell with capacity of 200 kN was utilized to determine the magnitude of loading, and the corresponding free end slip of each specimen was continuously recorded via a data-acquisition system.

The AE signals emitted from the debonding process of FRP bar with the hosting concrete cube were collected by a Micro-II digital system (manufactured by Physical Acoustics Corporation, Princeton, NJ, USA), as shown in Figure 3b. PAC R6a sensors, with an effective operation frequency ranging from 35–100 kHz, were adopted in this test. The R6a sensor has been widely used on metal and FRP structures due to its high sensitivity and low resonance frequency properties. The peak sensitivity (Ref V/(m/s)) of R6a sensors is 75 dB. Before the experiment, the pencil lead break testing will be done to ensure that the R6a sensors have high accuracy. The sampling rate is 1MHz. To shield the noise signals, the threshold was set as 45 dB. To improve the accuracy of linear location, PDT (peak definition time: ensures correct identification of the signal peak for risetime and peak amplitude measurements), HDT (hit definition time: ensures that each AE signal from the structure is reported as one and only one hit), and HLT (hit lockout time: inhibits the measurement of signals after the hit stored to avoid measuring reflection) were set as 300, 600, and 1000 µs respectively to reduce reflected waves. Two AE sensors were attached to the end faces of the bar for linear locating (see Figure 3c).

## 3. Detection Principles

The AE technique is used to collect and analyze the transient elastic wave caused by the rapid release of energy of materials, due to the process of deformation or fracture of the materials under stress [64,65]. As this phenomenon occurs inside materials, the elastic wave caused by fracture contained the fracture information of materials. Therefore, it is feasible to identify the damage process of the material by analyzing the elastic wave. In this paper, the AE signal strength and the frequency components were used to describe the debonding process. The signal strength is mathematically defined as the integral of the rectified voltage signal over the duration of the AE waveform packet. Compared to the other AE parameters (such as energy, amplitude), it was not only more sensitive but also had larger measuring ranges.

Furthermore, the linear location technique is applied in this investigation. It is evident that the debonding process between the reinforcement bars and SCC is available to release a lot of elastic waves. Those waves can propagate along the bars and is collected by AE sensors which are located at the two ends of the bars, as shown in Figure 3c. The arriving time *t*_top_ and *t*_bottom_ is defined as the time of the elastic wave reaching the top AE sensor and the bottom AE sensor, respectively. Compared to the arriving time of two AE sensors, the location of damage can be determined, as shown in Equation (1):

*L*_top_ = [(*t*_top_ − *t*_bottom_) *S* + *L*]/2, (1)

where *L*_top_ is the distance from the AE sensor which is mounted on the top of the bars; *S* is the elastic wave speed; and *L* is the total length of the bars. As a result, the damage characteristics in the debonding process are investigated through studying the damage location, the signal strength and the damage frequency. It is found that the distribution of the signal strength and the frequency through the reinforcing bars can be clearly presented in the AE method. In addition, the relationship of the location of signal strengths and the frequency of the damage signals between the testing time is also adopted to study the failure mechanism of the bonding behavior of FRP–SCC effectively.

## 4. Discussion of Test Results

### 4.1. Mechanical Properties

The samples were designated according to rebar type (BFRP, GFRP, and steel), bar diameter (12 mm and 20 mm), embedded length (40 mm, 80 mm and 120 mm), and fiber volume content (0.0%, 0.3% and 0.6%). For instance, the BFRP reinforced SCC sample cast with fiber content of 0.3%, bar diameter of 12 mm, and embedded length of 80 mm is designated as SCC-0.3%-BFRP-d12-80. A uniform distribution of bond stress within the embedded length is assumed, and the average bond stress is defined as:

τ = *P*/(*πdl*) (2)

where *P* is the pull-out force (kN), *d* is the nominal bar diameter (mm), and *l* is the embedded length (mm). The experimental results obtained from the direct pull-out tests, as well as the mode of failure, are summarized in Table 4. In this table, *P*_max_ is the maximum pull-out force, *τ*_max_ is the maximum average bond strength, *s*_fp_ is the slip value at the bond strength for the free end, and *τ*_re_ is the residual bond stress in the post peak phase. For all tested specimens, the average bond stress defined by Equation (2) and the corresponding slip at free end were evaluated, as shown in the following section.

#### 4.1.1. Effect of Rebar Type on Bonding Behavior

**Figure 4:**
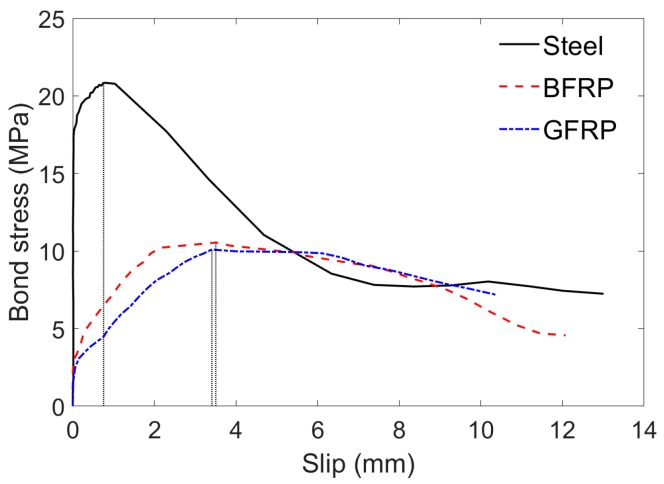
Bond stress versus slip at free end of specimens with different rebar types.

Figure 4 shows the relationship between bond stress and the slip obtained, respectively, for SCC-0.0%-BFRP-d12-80, SCC-0.0%-GFRP-d12-80 and SCC-0.0%-Steel-d12-80, which differed in rebar type only. As shown in Figure 4, the BFRP reinforced specimen had similar bonding behavior to that of the GFRP one. They provide, however, some 50% of bond strength compared with the steel reinforced specimen but with much greater slip values. This phenomenon is mainly caused by the fact that the elastic modulus of BFRP and GFRP bars are lower than that of the steel bar (Table 1), as also suggested by Caro et al. [29] and other scholars [21,22,23]. Moreover, there is no evident dropping stage for specimens reinforced with FRP bars, especially for the GFRP specimen. The residual bond stress ratio of the sand-coated GFRP bar used in this study was about 71.2%, approximately two times higher than that of ribbed steel bar (34.8%).

#### 4.1.2. Effect of Bar Diameter on Bonding Behavior

**Figure 5:**
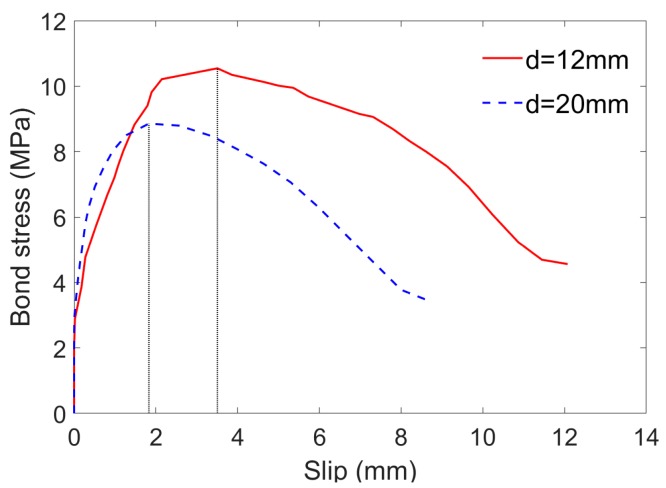
Bond stress versus slip at free end of specimens with different bar diameters.

The influence of bar diameter on bonding behavior can be inferred from specimens SCC-0.0%-BFRP-d12-80 and SCC-0.0%-BFRP-d20-80 (Table 4). The two specimens were almost identical except for bar diameters. The comparison of the bond stress-slip curves is plotted in Figure 5. As shown in Figure 5, the increase in bar diameter from 12 mm to 20 mm resulted in the decrease of bond strength by about 16%. Meanwhile, the corresponding slip value decreased by 48%. The experimental results confirm the tendency of larger bar diameters to develop lower bond strength. During the pull-out test, the larger the rebar diameter, the more serious the Poisson effect and the more obvious the shear lag phenomenon, which results in the lower bonding strength. The reasons for this phenomenon can also be found in the literature [21,24,25,26].

#### 4.1.3. Effect of Embedded Length on Bonding Behavior

**Figure 6:**
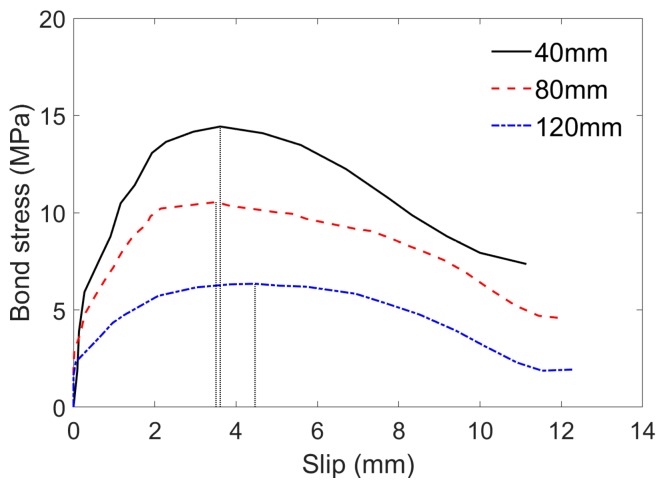
Bond stress versus slip at free end of specimens with different embedded lengths.

The bond stress-slip relationship for the BFRP bar specimens with different embedded lengths (40 mm, 80 mm, and 120 mm) are shown in Figure 6. Clearly, there is a decreasing trend of the maximum average bond stress with the increase in embedded length. During the pull-out test, the bond stress transfers gradually from the loaded end to the free end, having a non-linear distribution of bond stress along the embedment length. The nonlinear stress distribution is more evident in the case of larger embedment length, which results in a lower bond strength [27,28,29]. The nonlinear distribution of stress along the bar is further confirmed by AE method in this study.

#### 4.1.4. Effect of Fiber Volume Content on Bonding Behavior

**Figure 7:**
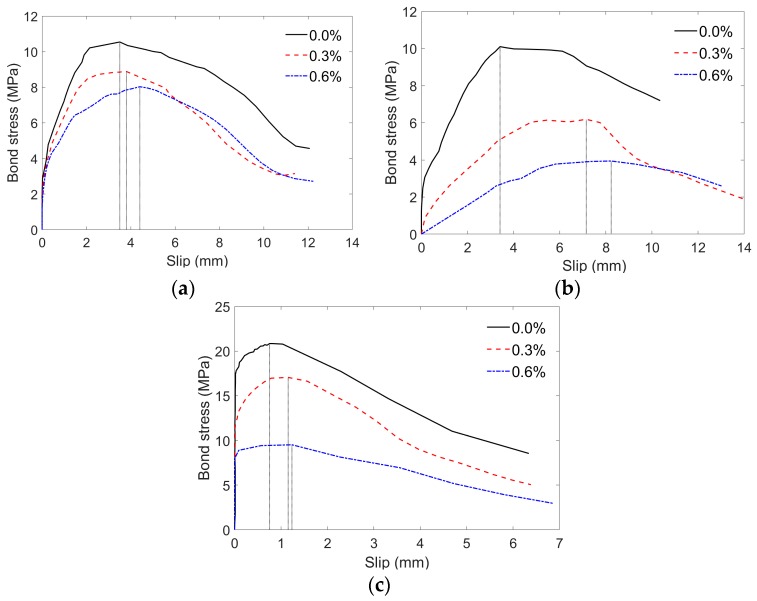
Bond stress versus slip at free end of specimens with different fiber volume contents: (**a**) Specimen reinforced with BFRP bars; (**b**) Specimen reinforced with GFRP bars; (**c**) Specimen reinforced with steel bars.

Figure 7 illustrates the influence of PP fiber volume contents on the bonding behavior of specimens reinforced with different types of bars (BFRP, GFRP and steel bar). As shown in Figure 7, higher PP fiber volume contents unexpectedly developed lower bonding strengths. Compared with the SCC-0.0%-BFRP-d12-80, SCC-0.0%-GFRP-d12-80, and SCC-0.0%-Steel-d12-80 specimens, the bonding strengths of SCC-0.6%-BFRP-d12-80, SCC-0.6%-GFRP-d12-80, and SCC-0.6%-Steel-d12-80 are decreased by 23.8%, 61.0%, and 54.4%, respectively. The non-uniform distribution of PP fibers in mortar increases the damage in the matrix and at the interface during the pull-out test, resulting in poorer friction resistance and a lower bonding strength.

### 4.2. AE Characteristic Parameters

**Figure 8:**
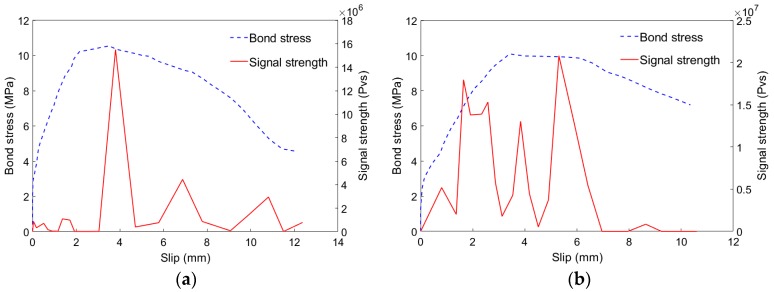

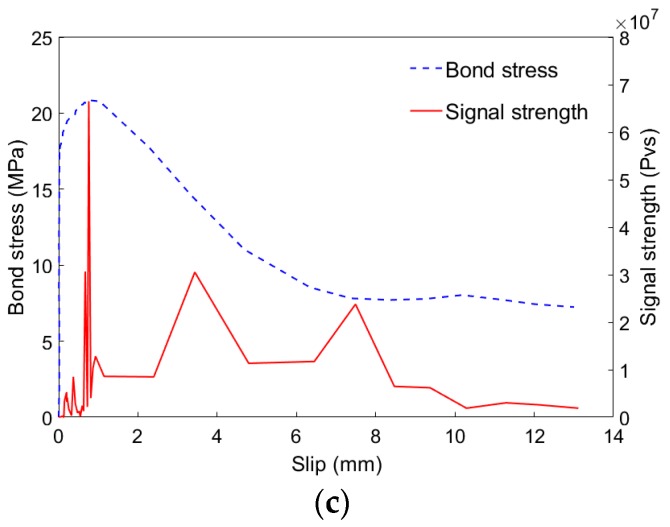
Corresponding relationship between signal strength and bond stress: (**a**) SCC-0.0%-BFRP-d12-80; (**b**) SCC-0.0%-GFRP-d12-80; (**c**) SCC-0.0%-Steel-d12-80.

Figure 8 shows a comparison of the AE signal strength and the bond stress. It can be found that the bond stress and the signal strength reached the maximum value almost at the same time. Meanwhile, the maximum value of signal strength in the steel bar was much larger than those of the BFRP and GFRP bars. Furthermore, there are some fluctuations in the AE signal strength curves. These fluctuations represent some damage which release lower energy. However, the stress-slip curves acquired by traditional testing methods cannot reflect these damages clearly. In addition, AE signals include the information of frequency and damage location, so it can reflect the debonding process more comprehensively and accurately.

**Figure 9:**
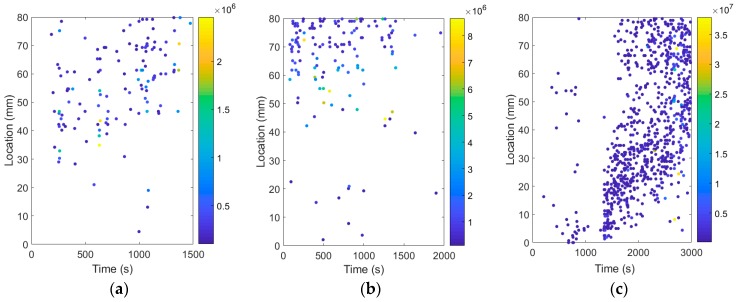
Variation tendency of damage location with time: (**a**) SCC-0.0%-BFRP-d12-80; (**b**) SCC-0.0%-GFRP-d12-80; (**c**) SCC-0.0%-Steel-d12-80.

Figure 9 illustrates the variation tendency of damage location with time. The Y-axis represents the location of the damage along the embedment length: every dot represents one AE event, and the color of dots represents the value of the signal strength. In the steel bar, as shown in Figure 9c, a few dots distributed in the loaded end and middle of the embedment length at the beginning of the test, and the signal strength of these dots was low. After debonding, the dots gradually spread to the free end of the steel bar and the dots with a larger signal strength occurred. This variation tendency is in agreement with the expectation that the debonding damage initiates at the loaded end, then propagates to the free end of the bars. By contrast, the damage location of the GFRP reinforced concrete specimens always distributed in the middle and free end of the bar, as shown in Figure 9b. In the BFRP reinforced concrete model, the dots also distributed in the middle and free end of the bar, but the dots with larger signal strength occurred in the middle of the bar firstly, and gradually spread to the free end of the bar, as shown in Figure 9a.

**Figure 10:**
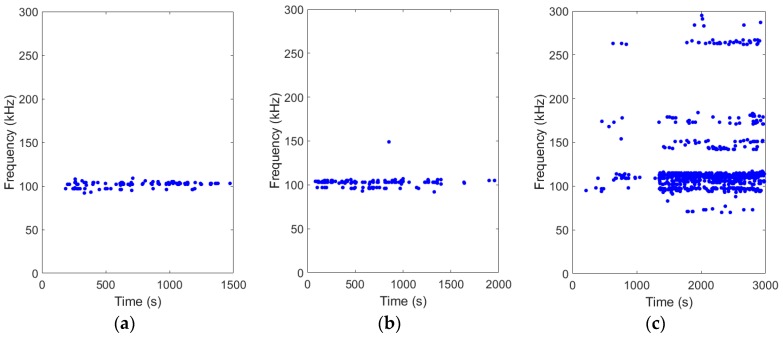
Frequency distribution of AE signal in the debonding process of specimens: (**a**) SCC-0.0%-BFRP-d12-80; (**b**) SCC-0.0%-GFRP-d12-80; (**c**) SCC-0.0%-Steel-d12-80.

Figure 10 illustrates the frequency distribution of AE signals in the debonding process of specimens reinforced with different types of bars (BFRP, GFRP, and steel bar). As shown in Figure 10, the frequency of debonding process in the BFRP bar and the GFRP bar was concentrated around 100kHz; however, that in the steel bar distributed in the around 100 kHZ, 140–155 kHz, 170–185 kHz and 260–295 kHz. It’s obvious that the frequency domain in the steel bar is wider than that in the FRP bar, particularly in the high frequency range. This can be attributed to the fact that the mechanical interlocking between the steel ribs and concrete paste is much stronger than that between the FRP bar and concrete paste. As a result, the debonding process generates more high-frequency signal in the steel bar than in the FRP bar.

## 5. Conclusions

The AE method was utilized to investigate the bond behavior between FRP/steel bars and self-compacting concrete in this study. A total of 36 pull-out tests with different bar types (BFRP, GFRP, and steel), bar diameters (12 mm and 20 mm), embedded lengths (40 mm, 80 mm, and 120 mm), and PP fiber contents (0.0%, 0.3%, and 0.6%) were performed. For each test, the average bond stress-slip relationship and the AE due to the debonding process were continuously measured. Based on the test results, the following conclusions are drawn.

The type of pull-out failure was observed for all the specimens in this study. Due to the low elastic modulus and the different surface treatment, the FRP reinforced specimens show a lower bond strength and a greater corresponding slip value than the steel reinforced specimens. The bond strength between bars and the hosting SCC decreases with the increase in the bar diameter due to the Poisson effect and the shear lag phenomenon. In general, the specimens with a shorter bar embedded length can achieve a higher level of average bond strength because of the non-linear distribution of stress along the bar. The non-uniform distribution of PP fibers may lead a poorer friction resistance at the interface during the pull-out test, thus resulting a lower bond strength.

The AE signal strength was consistent with the bond stress measured in each specimen, which can be used as a basis to monitor the bonding strength of FRP/steel bars in SCC. Meanwhile, the AE location technique revealed the non-linear distribution of bond stress along the bar, the initial location of damage and the debonding process of the pull-out tests can be captured by the proposed AE location technique. Moreover, it is revealed that the frequency components of the AE signals measured in the FRP and steel reinforced test specimens were significantly different. This phenomenon indicates that failure mode of the mechanical interlocking in the case of steel is stronger than the failure mode of interfacial friction in the FRP reinforced concrete models. As a result, the frequency released in the steel bar is generally higher than that in FRP reinforcing materials.

## Figures and Tables

**Table 1:** Properties of the reinforcement bars (Mean values).

Material Type	Diameter (mm)	Yield Strength (N/mm^2^)	Ultimate Strength (N/mm^2^)	Elastic Modulus (N/mm^2^)	Strain at Ultimate Strength (%)
BFRP	12	/	1032	48	2.2
BFRP	20	/	900	45	2.1
GFRP	12	/	1153	52	2.0
Steel	12	487	589	210	10.0

**Table 2:** Composition of the concrete mixture.

Components	Quantity ^1^ (kg)		
	SCC-0.0%	SCC-0.3%	SCC-0.6%
Cement 32.5R	13.68	15.12	18.00
Fly ash	18.24	20.16	24.00
Limestone powder	4.56	5.04	6.00
Fine river sand	55.68	55.68	55.68
Crushed granite	40.32	40.32	40.32
Water	12.77	13.55	15.60
Superplasticizer	0.073	0.081	0.096
Polypropylene fibers	0.00	0.169	0.352

^1^ SCC = self-compacting concrete; xx% = volume content of polypropylene fibers.

**Table 3:** Mechanical properties of the self-compacting concrete (SCC) batches (Mean values).

Batch Designation	Compressive Strength (N/mm^2^)	Tensile Strength (N/mm^2^)	Elastic Modulus (N/mm^2^)
SCC-0.0%	54.4	3.7	3.1 × 10^4^
SCC-0.3%	49.5	3.7	3.1 × 10^4^
SCC-0.6%	48.5	3.5	2.8 × 10^4^

**Table 4:** Bond results of the specimens.

Specimen Designation	*P*_max_ (kN)	*τ*_max_ (N/mm ^2^)	*s*_fp_ (mm)	*τ*_re_ (N/mm^2^)	*τ*_re_/*τ*_max_	Failure Mode ^1^
SCC-0.0%-BFRP-d12-40	21.76	14.43	3.61	7.36	51.0%	PO
SCC-0.0%-BFRP-d12-80	31.79	10.54	3.51	4.56	43.3%	PO
SCC-0.0%-BFRP-d12-120	28.73	6.35	4.47	1.93	30.5%	PO
SCC-0.0%-BFRP-d20-80	44.48	8.85	1.83	3.41	38.5%	PO
SCC-0.3%-BFRP-d12-80	26.81	8.89	3.81	3.15	35.5%	PO
SCC-0.6%-BFRP-d12-80	24.22	8.03	4.41	2.72	33.9%	PO
SCC-0.0%-GFRP-d12-80	30.46	10.10	3.41	7.19	71.2%	PO
SCC-0.3%-GFRP-d12-80	18.67	6.19	7.15	2.76	44.6%	PO
SCC-0.6%-GFRP-d12-80	11.88	3.94	8.23	2.60	65.8%	PO
SCC-0.0%-Steel-d12-80	62.85	20.84	0.76	7.25	34.8%	PO
SCC-0.3%-Steel-d12-80	51.42	17.05	1.16	5.05	29.6%	PO
SCC-0.6%-Steel-d12-80	28.68	9.51	1.24	2.96	31.1%	PO

^1^ PO = pull-out.
